# Family Physician–to–Hospital Specialist Electronic Consultation and Access to Hospital Care: A Systematic Review

**DOI:** 10.1001/jamanetworkopen.2023.51623

**Published:** 2024-01-12

**Authors:** Ken M. M. Peeters, Loïs A. M. Reichel, Dennis M. J. Muris, Jochen W. L. Cals

**Affiliations:** 1 Department of Family Medicine, Care and Public Health Research Institute (CAPHRI), Maastricht University, Maastricht, the Netherlands; 2 Zuyderland Medical Centre, Sittard, the Netherlands; 3 Omnes Medical Coordinating Centre for Diagnostics and Innovation, Sittard, the Netherlands; 4 Public Health Service South Limburg, Heerlen, the Netherlands

## Abstract

**Importance:**

Globally, health care systems face challenges in managing health care costs while maintaining access to hospital care, quality of care, and a good work balance for caregivers. Electronic consultations (e-consultations)—defined as asynchronous, consultative communication between family physicians and hospital specialists—may offer advantages to face these challenges.

**Objective:**

To provide a quantitative synthesis of the association of e-consultation with access to hospital care and the avoidance of hospital referrals.

**Evidence Review:**

A systematic search through PubMed, MEDLINE, and Embase was conducted. Eligible studies included original research studies published from January 2010 to March 2023 in English, Dutch, or German that reported on outcomes associated with access to hospital care and the avoidance of hospital referrals. Reference lists of included articles were searched for additional studies. Grading of Recommendations, Assessment, Development, and Evaluations (GRADE) scores were assigned to assess quality of evidence.

**Findings:**

The search strategy resulted in 583 records, of which 72 studies were eligible for data extraction after applying exclusion criteria. Most studies were observational, focused on multispecialty services, and were performed in either Canada or the US. Outcomes on access to hospital care and the avoidance of referrals indicated that e-consultation was associated with improved access to hospital care and an increase in avoided referrals to the hospital specialist, although outcomes greatly differed across studies. GRADE scores were low or very low across studies.

**Conclusions and Relevance:**

In this systematic review of the association of e-consultation with access to hospital care and the avoidance of hospital referrals, results indicated that the use of e-consultation has greatly increased over the years. Although e-consultation was associated with improved access to hospital care and avoidance of hospital referrals, it was hard to draw a conclusion about these outcomes due to heterogeneity and lack of high-quality evidence (eg, from randomized clinical trials). Nevertheless, these results suggest that e-consultation seems to be a promising digital health care implementation, but more rigorous studies are needed; nonrandomized trial designs should be used, and appropriate outcomes should be chosen in future research on this topic.

## Key Points

**Question:**

Is electronic consultation (e-consultation) associated with access to care and the avoidance of hospital referrals, and what is the quality of the evidence regarding these outcomes?

**Findings:**

In this systematic review of 72 studies (majority multispecialty and performed in North America) e-consultation was associated with improved access to hospital care and an increase in avoided referrals to the hospital specialist. However, Grading of Recommendations, Assessment, Development, and Evaluations scores were low for these studies.

**Meaning:**

These findings suggest e-consultation is associated with improved access to care and avoiding unnecessary hospital referrals; however, it is hard to draw conclusions due to heterogeneity and lack of high-quality evidence.

## Teaser

This systematic review analyzes electronic consultations (e-consultations) between family physicians and hospital specialists and the association with access to hospital care and referrals.

## Introduction

Electronic consultations (e-consultations) between family physicians (FPs), also known as primary care physicians, and hospital specialists are an emerging health innovation and a form of asynchronous digital interdisciplinary communication. An FP initiates an e-consultation through a secured digital platform in which the FP discloses a specific clinical question to a hospital specialist or seeks advice about the care of a particular patient, after which the specialist submits an answer to the FP.^1,2^ Globally, health care systems face challenges in managing health care costs while maintaining access to hospital care, quality of care, and a good work balance for caregivers. E-consultations may offer advantages to face these challenges.^1,3^ These advantages include fewer disruptions in clinical work compared with telephone consultations, allowing physicians to initiate or respond to an e-consultation when they are available. Furthermore, it allows for the digital and direct sharing of patient information, such as recent diagnostic evaluations or medical history, including documentation of communication between both physicians in the patient record.^4,5,6^ Previous research^7^ has shown that the majority of studies on e-consultation were conducted in the US and Canada, with most studies focusing on multispecialty services.

Previous systematic reviews^7,8^ analyzed studies of e-consultations between FPs and hospital specialists using a narrative synthesis approach, including an overview of its association with population health. Yet, from the perspective of the 2 people in the FP’s consultation room (ie, the FP and the patient), the questions that are most relevant when contemplating using an e-consultation to contact a hospital specialist are: (1) does it provide the patient with timely access to hospital care? and (2) does it enable the FP to continue to provide appropriate and well-informed care without the patient needing to visit the hospital outpatient department?

The focus of this review is to provide a quantitative synthesis of the outcomes of these questions (ie, access to hospital care and the avoidance of hospital referrals). We aimed to answer the following questions: (1) what is the association of FP–to–hospital specialist e-consultation with access to care and the avoidance of hospital referrals? and (2) what is the quality of the evidence regarding these outcome measures?

## Methods

### Search Strategy

This systematic review followed the Preferred Reporting Items for Systematic Reviews and Meta-Analyses (PRISMA) reporting guideline, and did not require institutional review board approval because it did not constitute human participants research in accordance with the Common Rule. We performed a systematic review of FP–to–hospital specialist e-consultations, including an assessment of study quality and quantitative synthesis of outcomes; the quantitative synthesis assessed the association of e-consultations with a patient's access to health care and the avoidance of hospital referrals. We defined an e-consultation as an asynchronous, consultative communication between FPs and hospital specialists within a shared electronic health record that seeks clarification or guidance regarding clinical care for a patient not already under treatment by a hospital specialist. We systematically searched PubMed, MEDLINE, and Embase. We included studies published in English, Dutch, or German from January 2010 to March 2023. Furthermore, we searched reference lists of included articles for additional studies. eAppendix 1 in Supplement 1 shows our systematic review protocol (including search strategy), which was not registered. The protocol and search strategy were discussed with the research team.

### Screening Process

We screened the studies using Covidence systematic review software (Veritas Health Innovation) for article type, relevance, and outcome measures. We only included original research studies and therefore excluded opinion papers, policy papers, guidelines, preprints, protocols, reviews, notes, editorials, letters, and abstracts. Furthermore, we excluded studies evaluating e-consultations that did not fit our previously mentioned definition (eg, patient-doctor e-consultations, unsolicited e-consultations, video consultations, email consultations, 1-way consultations, patient-clinician modalities, electronic referrals, online discussion forums, and studies that did not study the effect of e-consultations separately). We also excluded studies about teledermatology because we viewed this as a stand-alone type of e-consultation, and multiple reviews have reported on outcomes of the specific service. We excluded studies that did not contain any of the outcome measurements on access to care or the avoidance of hospital referrals.

Two reviewers (K.P. and D.M.) screened the abstracts and full texts for relevancy. We used group discussion to reach a consensus if there was a disagreement about the relevance of a study, and if no consensus was reached, a third reviewer (J.C.) was available as the decisive rater.

### Outcomes on Access to Care and the Avoidance of Hospital Referrals

In Supplement 1, eAppendix 2 shows the included outcome measurements. Access to care comprised 3 outcome measurements: response time, time spent answering the e-consultation by the specialist, and wait time for the patient to receive hospital consultation, either electronically or live at the outpatient department.

Because studies used different methods to report hospital referrals, we aimed to hierarchically categorize the studies beginning with the most solid evidence; we first used the only direct measurement of avoided referrals: hospital visits. Beyond this direct measurement, we had 3 indirect measurement methods for possible referrals: referral requests by FPs, postconsult surveys by FPs, and referral recommendations by hospital specialists. FP-reported referrals are an indirect measurement because they do not account for no-shows and cancellations and thus did not tell us anything about the actual hospital visits. A postconsult survey is a survey that the FP completes after reading the specialist's answer in the e-consultation. In this survey, the FP clicks 1 of the following scenarios (Table 1):

1. Avoided referral: a referral was originally contemplated but is now avoided at this stage.
2. Referral still needed: a referral was originally contemplated and is still needed.
3. Referral still not needed: a referral was not originally contemplated and is still not needed.
4. Extra referral: a referral was not originally contemplated but the e-consultation resulted in a referral being initiated.

These postconsult surveys do not provide the actual number of requested referrals. There might be a discrepancy between the number of FPs stating that a referral is needed and the number of actual FP referrals. Lastly, an FP might ignore a hospital specialist's referral advice, which is also an indirect measurement of avoided referrals.

**Table 1.** Cross-Table Showing the 4 Different Referral Scenarios, Dependent on the FP Having or Not Having the Intention to Refer the Patient if the E-Consultation Was Not Available and the Actual Referral of the Patient

FP intention to refer	Patient actually referred		Total
	Yes	No	
Yes	Referral still needed	Avoided referral	Total number where FP intended to refer
No	Extra referral	Referral still not needed	Total number where FP did not intend to refer
Total	Total referred	Total not referred	Total number of patients

Abbreviation: FP, family physician.

### Data Extraction 

Two reviewers (K.P. and L.R.) extracted the data from Covidence. Data extracted included title, author, country, year, aim, study design, sample size, main outcomes, and findings.

### Quality of the Evidence

Two independent raters (K.P. and L.R.) assigned quality ratings for each publication using the Grading of Recommendations, Assessment, Development, and Evaluations (GRADE) score.^9,10^ Typically, randomized clinical trials (RCTs) receive a high GRADE score, whereas observational studies receive a low GRADE score. The quality of the evidence can be downgraded if methodological flaws exist, and the quality of evidence can be upgraded when high rigor and large effect sizes exist.^9^ We used group discussion to reach a consensus if there was a disagreement about the quality of evidence, and if no consensus was reached, a third rater (J.C.) was available as the decisive rater.

### Statistical Analysis

We conducted a quantitative synthesis of included studies. We used the Meta-Essentials software package version 1.4 (Erasmus Research Institute of Management) to see if an estimate of effect size was possible by assessing heterogeneity using the *I^2^* statistic.^11^ If studies reported both medians and means, we reported the medians. Measurements were excluded if only percentage ranges instead of values were reported.

## Results

The search strategy on PubMed, MEDLINE, and Embase resulted in 583 records. After the abstract screening, we removed 402 records after applying exclusion criteria. We screened 181 full-text articles, of which we excluded 108 records after applying exclusion criteria. A total of 72 studies^2,3,12,13,14,15,16,17,18,19,20,21,22,23,24,25,26,27,28,29,30,31,32,33,34,35,36,37,38,39,40,41,42,43,44,45,46,47,48,49,50,51,52,53,54,55,56,57,58,59,60,61,62,63,64,65,66,67,68,69,70,71,72,73,74,75,76,77,78,79,80,81^ were eligible for data extraction (Figure).

**Figure.**
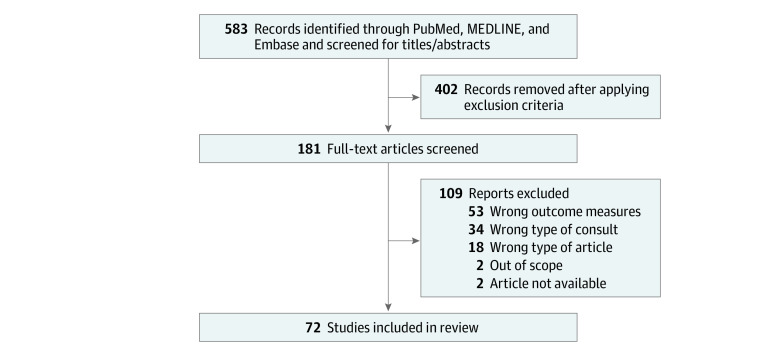
Flow Diagram

 Of the 72 studies, 34 (47%) were conducted in the US,^12,14,19,29,31,32,34,35,36,37,38,40,44,46,47,48,49,53,54,57,61,62,63,66,67,68,69,70,71,72,73,78,79^ 31 (43%) in Canada,^3,13,15,16,17,18,20,21,22,23,24,25,26,27,28,30,33,39,41,42,43,45,50,51,52,55,58,75,76,77^and 7 (10%) in the rest of the world^2,56,59,60,64,65,81^ (Table 2). Of the 72 studies, 31 (43%) focused on multispecialty services,^3,16,18,19,21,22,24,25,26,27,28,30,32,33,35,38,43,44,51,52,57,66,67,68,69,70,76,77,80^ and 14 (19%) focused on internal medicine or a subspecialty of internal medicine.^2,12,14,17,23,31,39,40,50,53,59,63,71,79^ The number of included e-consultations per study ranged from 54 to 3 177 998. Of all 72 studies, 2 (3%) were RCTs,^34,80^ 4 (5%) were nonrandomized trials,^12,37,61,64^ and 66 (92%) were observational.^2,3,13,14,15,16,17,18,19,20,21,22,23,24,25,26,27,28,29,30,31,32,33,35,36,38,39,40,41,42,43,44,45,46,47,48,49,50,51,52,53,54,55,56,57,58,59,60,62,63,65,66,67,68,69,70,71,72,73,74,75,76,77,78,79,81^ The most frequently mentioned reasons for implementing the e-consultation were improving access to hospital care (63 of 72 studies^3,12,13,14,15,16,17,18,19,20,21,22,23,24,25,26,27,28,29,30,31,32,33,34,35,36,37,38,39,41,42,43,44,45,46,47,49,50,51,52,54,55,56,57,58,59,60,63,65,66,67,68,70,71,73,74,75,76,77,78,79,80^ [88%]), avoiding hospital referrals (28 of 72 studies^14,17,18,19,21,23,28,31,36,38,39,40,53,56,57,58,59,60,63,64,65,66,67,68,69,71,76,77^ [38%]), and enhancing clinician communication (9 of 72 studies^34,38,57,61,62,69,71,76,78^ [13%]). Reasons for avoiding hospital referrals included (1) reducing unnecessary referrals (28 of 72 studies^14,17,18,19,21,23,28,31,36,38,39,40,53,56,57,58,59,60,63,64,65,66,67,68,69,71,76,77^ [38%]), for example, during the COVID-19 pandemic or for patients unable to travel to the hospital, and (2) controlling health care costs (7 of 72 studies^2,14,28,48,69,70,78^ [10%]). eAppendix 3 in Supplement 1 shows that the GRADE score for the avoided referrals outcome measurement was low. The GRADE scores for the outcome measurements of response time, time spent answering e-consultation by specialist, and wait time were very low. *I^2^* was 100%, showing a large degree of heterogeneity.

**Table 2.** Characteristics of Included Studies by Author in Alphabetical Order

Study	Country	Design	Consultations, No.	Specialty	Main outcomes	Reason for implementing e-consultation
Anderson et al,^12^ 2020	US	Nonrandomized trial	Pre-EC: 365; post-EC: 469	Endocrinology	Response time; wait time; referrals	Improve access to hospital care
Archibald et al,^13^ 2018	Canada	Observational	169	Psychiatry	Response time; referrals	Improve access to hospital care
Avery et al,^47^ 2021	US	Observational	343	Psychiatry	Response time; referrals	Improve access to hospital care
Bauer et al,^48^ 2020	US	Observational	4833	Cardiology	Response time; time spent answering	Control health care costs
Bhavsar et al,^49^ 2019	US	Observational	187	Hepatology	Response time; wait time; referrals	Improve access to hospital care
Burwick et al,^14^ 2018	US	Observational	3217	Hematology	Response time	Control health care costs; reduce unnecessary referrals (and save travel time); improve access to hospital care
Chang et al,^15^ 2020	Canada	Observational	212	Pediatric orthopedic surgery	Response time; referrals	Improve access to hospital care
Chittle et al,^62^ 2015	US	Observational	54	Vascular surgery	Referrals	Enhance clinician communication
de Man et al,^16^ 2019	Canada	Observational	291	Multiple	Response time	Improve access to hospital care
Dosani et al,^63^ 2022	US	Observational	7664	Hematology	Wait time; Referrals	Improve access to hospital care; reduce unnecessary referrals (triggered by COVID-19)
Fernández-Prada et al,^64^ 2020	Spain	Nonrandomized trial	137	Vaccinology	Referrals	Reduce unnecessary referrals (triggered by COVID-19)
Fogel et al,^17^ 2017	Canada	Observational	436	Hematology	Response time; referrals	Improve access to hospital care reduce unnecessary referrals
Fulford et al,^72^ 2016	US	Observational	42	Psychiatry	Referrals	Improve access to hospital care
Guglani et al,^18^ 2022	Canada	Observational	26 121	Multiple	Response time; time spent answering; referrals	Improve access to hospital care; reduce unnecessary referrals
Gupte et al,^19^ 2016	US	Observational	7097	Multiple	Response time	Improve access to hospital care; reduce unnecessary referrals
Hadden et al,^20^ 2022	Canada	Observational	564	Orthopedic surgery	Response time; time spent answering; referrals	Improve access to hospital care
Helmer-Smith et al^21^, 2020	Canada	Observational	64	Multiple	Response time; time spent answering; referrals	Improve access to hospital care; reduce unnecessary referrals (impaired patients unable to travel)
Johnston et al,^50^ 2017	Canada	Observational	85	Hematology and oncology	Response time; referrals	Improve access to hospital care
Keely et al,^3^ 2013	Canada	Observational	406	Multiple	Response time; referrals	Improve access to hospital care
Keely et al,^58^ 2020	Canada	Observational	11 985	Multiple	Time spent answering	Improve access to hospital care; reduce unnecessary referrals
Kim et al,^78^ 2019	US	Observational	393	Cardiology	Referrals	Control health care costs; improve access to hospital care; enhance clinician communication
Kinberg et al,^61^ 2021	US	Nonrandomized trial	Pre-EC: 2931; post-EC 3686	Otolaryngology	Wait time; referrals	Enhance clinician communication
Lai et al,^22^ 2018	Canada	Observational	1064	Multiple	Response time; Referrals	Improve access to hospital care
Lai et al,^23^ 2022	Canada	Observational	276	Internal medicine	Response time; time spent answering; referrals	Improve access to hospital care; reduce unnecessary referrals
Lee et al,^79^ 2021	US	Observational	120	Rheumatology	Referrals	Improve access to hospital care
Liddy et al,^51^ 2013	Canada	Observational	77	Multiple	Response time	Improve access to hospital care
Liddy et al,^24^ 2017	Canada	Observational	594	Multiple	Response time; referrals	Improve access to hospital care
Liddy et al,^25^ 2018	Canada	Observational	14 105	Multiple	Response time; referrals	Improve access to hospital care
Liddy et al^52^ 2019	Canada	Observational	Range: 96-6885	Multiple	Response time; referrals	Improve access to hospital care
Liddy et al,^80^ 2019	Canada	Randomized clinical trial	44 066 (22 079 e-consultation vs 21 987 control group)	Multiple	Referrals	Improve access to hospital care
Liddy et al,^26^ 2019	Canada	Observational	301	Multiple	Response time; referrals	Improve access to hospital care
Liddy et al,^27^ 2020	Canada	Observational	3233	Multiple	Response time; referrals	Improve access to hospital care
Liddy et al,^28^ 2022	Canada	Observational	60 474	Multiple	Response time; time spent answering; referrals	Improve access to hospital care; reduce unnecessary referrals; control health care costs
Lowenstein et al,^29^ 2017	US	Observational	50	Psychiatry	Response time; wait time; referrals	Improve access to hospital care
Lu et al,^74^ 2019	US	Observational	111	Psychiatry	Referrals	Improve access to hospital care
Mann et al,^65^ 2018	New Zealand	Observational	4738	Gynecology	Referrals	Improve access to hospital care; reduce unnecessary referrals
McKellips et al,^30^ 2017	Canada	Observational	127	Multiple	Response time	Improve access to hospital care
Mendu et al,^31^ 2016	US	Observational	74	Nephrology	Response time; time spent answering; referrals	Improve access to hospital care; reduce unnecessary referrals
Muris et al,^2^ 2020	Netherlands	Observational	1250	Internal Medicine	Wait time; referrals	Control health care costs
Mustafa et al,^32^ 2020	US	Observational	109	Multiple	Response time; wait time; time spent answering; referrals	Improve access to hospital care
Nabelsi et al,^33^ 2019	Canada	Observational	1016	Multiple	Referrals; response time	Improve access to hospital care
North et al,^66^ 2015	US	Observational	5334	Multiple	Referrals	Improve access to hospital care; reduce unnecessary referrals
Olayiwola et al,^34^ 2016	US	Randomized clinical trial	590	Cardiology	Response time; wait time; referrals	Improve access to hospital care; enhance clinician communication
Olayiwola et al,^35^ 2019	US	Observational	3872	Multiple	Referrals	Improve access to hospital care
Olayiwola et al,^81^ 2020	Nigeria	Observational	23	Multiple	Response time	Improve access to hospital care
Patel et al,^53^ 2019	US	Observational	281	Endocrinology	Response time; wait time	Reduce unnecessary referrals (patients unable to travel)
Pecina et al,^67^ 2016	US	Observational	220	Multiple	Response time; referrals	Improve access to hospital care; reduce unnecessary referrals
Pecina et al,^68^ 2016	US	Observational	5115	Multiple	Referrals	Improve access to hospital care; reduce unnecessary referrals
Phadke et al,^36^ 2019	US	Observational	306	Immunology	Response time; time spent answering; wait time; referrals	Improve access to hospital care; reduce unnecessary referrals
Porto et al,^37^ 2021	US	Nonrandomized trial	Pre-EC:680; post-EC: 749	Pediatrics	Response time; referrals	Improve access to hospital care
Rea et al,^38^ 2018	US	Observational	510	Multiple	Response time; wait time; referrals	Improve access to hospital care; reduce unnecessary referrals; enhance clinician communication
Rey-Aldana et al,^60^ 2021	Spain	Observational	29 321	Cardiology	Wait time; referrals	Improve access to hospital care; reduce unnecessary referrals
Rostom et al,^39^ 2018	Canada	Observational	225	Rheumatology	Response time; referrals	Improve access to hospital care; reduce unnecessary referrals
Russell et al,^59^ 2021	Australia	Observational	40	Endocrinology	Time spent answering; referrals	Improve access to hospital care; reduce unnecessary referrals
Saxon et al,^69^ 2021	US	Observational	3 117 998	Multiple	Referrals	Reduce unnecessary referrals; control health care costs; enhance clinician communication
Schettini et al,^40^ 2019	US	Observational	853	Nephrology	Response time; time spent answering; wait time; referrals	Reduce unnecessary referrals
Shehata et al,^75^ 2016	Canada	Observational	394	Obstetrics and gynecology	Referral	Improve access to hospital care
Shipherd et al,^54^ 2016	US	Observational	303	Transgender care	Response time; time spent answering	Improve access to hospital care
Singh et al,^41^ 2021	Canada	Observational	62	Transgender care	Response time; time spent answering; referrals	Improve access to hospital care
Singh et al,^42^ 2022	Canada	Observational	289	COVID-19	Response time; time spent answering; referrals	Improve access to hospital care
Singh et al,^43^ 2022	Canada	Observational	Total: 63 090; postconsultation survey: 30 320	Multiple	Response time; time spent answering; referrals	Improve access to hospital care
Stendahl et al,^44^ 2021	US	Observational	351	Multiple	Response time	Improve access to hospital care
Thompson et al,^70^ 2021	US	Observational	>7000	Multiple	Referrals	Improve access to hospital care; control health care costs
Tran et al,^76^ 2016	Canada	Observational	1080	Multiple	Referrals	Improve access to hospital care; reduce unnecessary referrals; enhance clinician communication
Ulloa et al,^73^ 2017	US	Observational	1743	General surgery	Referrals	Improve access to hospital care
Venkatesh et al,^71^ 2019	US	Observational	144	Gastroenterology	Referrals	Improve access to hospital care; reduce unnecessary referrals; enhance clinician communication
Walker et al,^77^ 2020	Canada	Observational	302	Multiple	Referrals	Improve access to hospital care; reduce unnecessary referrals
Wang et al,^55^ 2021	Canada	Observational	329	Obstetrics and gynecology	Response time; referrals	Improve access to hospital care
Williams et al,^56^ 2012	Ireland	Observational	710	Neurology	Response time; referrals	Improve access to hospital care; reduce unnecessary referrals
Witherspoon et al,^45^ 2017	Canada	Observational	190	Urology	Response time; referrals	Improve access to hospital care
Wrenn et al,^57^ 2017	US	Observational	200	Multiple	Response time; referrals	Improve access to hospital care; reduce unnecessary referrals; enhance clinician communication
Xu et al,^46^ 2022	US	Observational	80	Neuroophthalmology	Response time; referrals	Improve access to hospital care

Abbreviation: EC, electronic consultation.

### Access to Care

#### Access to Specialist Input

We found 48 studies reporting the response time, of which 36 (75%) reported the median,^3,12,13,14,15,16,17,18,19,20,21,22,23,24,25,26,27,28,29,30,31,32,33,34,35,36,37,38,39,40,41,42,43,44,45,46^ and 12 (25%) reported the mean.^33,47,48,49,50,51,52,53,54,55,56,57^ The median (IQR) response time across studies ranged from 1 hour (0.5-2.0 hours) to 39 (not available [NA]) days. The mean (SD) response time ranged from 3.5 (NA) hours to 16.7 (NA) days.

#### Time Spent Answering E-Consultation by Specialist

A total of 16 studies reported the time spent answering an e-consultation by specialists, including 7 (43.8%) that reported the median^21,28,31,32,36,43,58^ and 9 (56.3%) that reported the mean.^18,20,23,40,41,42,48,54,59^ The median (IQR) times ranged from 5 (NA) minutes to 15 (10-15) minutes. The mean (SD) times ranged from 14 (NA) minutes to 78 (63-94) minutes.

#### Wait Times for Face-to-Face Clinical Visits

We found 11 studies reporting on wait times for face-to-face clinical visits. The definition of wait time across studies was poorly defined. Of these 11 studies, 7 (64%) reported the median^12,29,32,34,36,40,60^ and 4 (36%) reported the mean.^38,49,53,61^ The median (IQR) wait time ranged from 7 (3-19) hours to 107 (NA) days and the mean (SD) wait time ranged from 34 (NA) days to 77 (NA) days. Anderson et al^12^ reported a decrease of 8 days in the median (IQR) wait time after implementing e-consultation (115 [NA] days vs 107 [NA] days; P > .05), and Phadke et al^36^ reported a decrease in the median wait time of 1.5 days (22.5 [20.8-24.0] days vs 21.0 [19.0-23.0] days; *P* < .05) after implementing the e-consultation. Olayiwola et al^34^ reported a 5-day increase in the median (IQR) wait time (24 [NA] days vs 29 [NA] days). Kinberg et al^61^ reported a decrease of 18 days in the mean (SD) wait time (61 [NA] days vs 43 [NA] days; P = .003), and Rea et al^38^ reported a decrease of 14 days in the mean (SD) wait time (48 [NA] days vs 34 [NA] days; *P* < .001).

### Avoidance of Hospital Referrals

We found 57 studies reporting on the percentage of patients who were not referred following an e-consultation (Table 3). Of these 57 studies, 20 (35%) reported hospital visits, showing percentages of patients not referred between 18.0% (92 of 510 referrals) and 97.5% (6825 of 7000 referrals).^2,32,37,38,40,49,50,57,60,61,62,63,64,65,66,67,68,69,70,71^ Authors described FP-reported referrals in 5 studies (9%), showing percentages of patients not referred between 19.0% (135 of 710 patients) and 84.7% (194 of 229 referrals).^12,36,56,72,73^ Postconsult FP surveys were used in 25 studies (44%), showing percentages of patients not referred between 26.3% (1018 of 3872 patients) and 95.0% (275 of 289 patients).^3,13,15,17,21,22,23,24,25,26,28,33,35,39,41,42,43,45,52,55,59,74,75,76,77^ Finally, 7 studies (12%) reported recommended referrals by specialists, showing percentages of patients not referred between 18.0% (22 of 120 patients) and 93.0% (319 of 343 patients).^29,31,34,46,47,78,79^ Kinberg et al^61^ reported an increase of 25 percentage points for patients not referred after implementation of e-consultations (410 of 2931 patients not referred before implementation [14%] vs 1438 of 3686 patients not referred after implementation [39%]; *P* <.001). Lu et al^74^ reported an increase of 24.0% for patients not referred after implementation of the e-consultation, and Mann et al^65^ reported an increase of 18.2%. In an RCT, Liddy et al,^80^ reported a 6% greater reduction in referrals in the intervention group compared with the control group, and a 7% relative reduction after adjusting for covariates, but these findings were not significant.

**Table 3.** The Percentage of Patients Not Referred, Time Span for Which Referrals Were Recorded and the Method of Measuring Avoided Referrals

Study	No. of patients not referred, No./No. of electronic consulations (%)	Time span	Method of measuring avoided referrals
Bhavsar et al,^49^ 2019	143/187 (76.5)	NA	Hospital visits
Chittle et al,^62^ 2015	46/54 (85.2)	90 d	Hospital visits
Dosani et al,^63^ 2022	6361/7664 (83.0)	1 y	Hospital visits
Fernández-Prada et al,^64^ 2020^a^	122/137 (89.1)	NA	Hospital visits
Johnston et al,^50^ 2017	65/85 (76.5)	NA	Hospital visits and postconsult survey
Kinberg et al,^61^ 2021^a^	1437/3686 (39.0)	NA	Hospital visits
Mann et al,^65^ 2018	4050/4738 (85.5)	6 mo	Hospital visits
Muris et al,^2^ 2020	1015/1250 (81.2)	3 mo	Hospital visits and postconsult survey
Mustafa et al,^32^ 2020	78/109 (71.6)	NA	Hospital visits
North et al,^66^ 2015	4389/5334 (82.3)	90 d	Hospital visits
Pecina et al,^67^ 2016	167/220 (76.0)	6 mo	Hospital visits
Pecina et al,^68^ 2016	4567/5115 (89.3)	28 d	Hospital visits
Porto et al,^37^ 2021^a^	92/176 (52.3)	NA	Hospital visits
Rea et al,^38^ 2018	91/510 (18.0)	6 mo	Hospital visits
Rey-Aldana et al,^60^ 2021	6274/29 321 (21.4)	NA	Hospital visits
Saxon et al,^69^ 2021	2 447 628/3 117 998 (78.5)	12 mo	Hospital visits
Schettini et al,^40^ 2019	575/853 (67.5)	NA	Hospital visits
Thompson et al,^70^ 2021	6825/7000 (97.5)	30 d	Hospital visits
Venkatesh et al,^71^ 2019	117/144 (81.3)	90 d	Hospital visits
Wrenn et al,^57^ 2017	172/200 (86.0)	6 mo	Hospital visits
Anderson et al,^12^ 2020^a^	193/229 (84.7)	NA	FP-reported referral
Fulford et al,^72^ 2016	19/42 (45.3)	NA	FP-reported referral
Phadke et al,^36^ 2019	165/306 (54.2)	NA	FP-reported referral
Ulloa et al,^73^ 2017	488/1743 (28.0)	NA	FP-reported referral
Williams et al,^56^ 2012	134/710 (19.0)	NA	FP-reported referral
Archibald et al,^13^ 2018	119/169 (71.0)	NA	Postconsult survey
Chang et al,^15^ 2020	144/212 (68.0)	NA	Postconsult survey
Fogel et al,^17^ 2017	289/436 (66.5)	NA	Postconsult survey
Helmer-Smith et al,^21^ 2020	50/64 (79.0)	NA	Postconsult survey
Keely et al,^3^ 2013	292/406 (72.0)	NA	Postconsult survey
Lai et al,^22^ 2018	744/1064 (70.0)	NA	Postconsult survey
Lai et al,^23^ 2022	110/276 (40.0)	NA	Postconsult survey
Liddy et al,^24^ 2017	457/594 (77.0)	NA	Postconsult survey
Liddy et al,^25^ 2018	9520/14105 (67.5)	NA	Postconsult survey
Liddy et al,^52^ 2019 (1)	6669/9087 (73.4)	NA	Postconsult survey
Liddy et al,^26^ 2019 (3)	174/301 (58.0)	NA	Postconsult survey
Liddy et al,^28^ 2022	47 169/60 474 (78.0)	NA	Postconsult survey
Lu et al,^74^ 2019	61/111 (55.6)	12 mo	Postconsult survey
Nabelsi et al,^33^ 2019	701/1016 (69.0)	NA	Postconsult survey
Olayiwola et al,^35^ 2019	1018/3872 (26.3)	NA	Postconsult survey
Rostom et al,^39^ 2018	146/225 (65.0)	NA	Postconsult survey
Russell et al,^59^ 2021	27/40 (69.0)	NA	Postconsult survey
Shehata et al,^75^ 2016	300/394 (76.2)	NA	Postconsult survey
Singh et al,^41^ 2021	50/62 (82.0)	NA	Postconsult survey
Singh et al,^42^ 2022 (1)	274/289 (95.0)	NA	Postconsult survey
Singh et al,^43^ 2022 (2)	23 649/30 320 (78.0)	NA	Postconsult survey
Tran et al,^76^ 2016	436/1080 (40.4)	NA	Postconsult survey
Walker et al, ^77^ 2020	208/302 (69.0)	NA	Postconsult survey
Wang et al,^55^ 2021	276/329 (84.0)	NA	Postconsult survey
Witherspoon et al,^45^ 2017	104/190 (55.0)	NA	Postconsult survey
Avery et al,^47^ 2021	318/343 (93.0)	NA	Recommended by specialist
Kim et al,^78^ 2019	348/393 (88.8)	NA	Recommended by specialist
Lee et al,^79^ 2021	21/120 (18.0)	NA	Recommended by specialist
Lowenstein et al,^29^ 2017	37/50 (74.0)	NA	Recommended by specialist
Mendu et al,^31^ 2016	57/74 (78.0)	NA	Recommended by specialist
Olayiwola et al,^34^ 2016^b^	408/590 (69.2)	NA	Recommended by specialist
Xu et al,^46^ 2022	56/80 (70.0)	NA	Recommended by specialist

Abbreviations: FP, family physician; NA, not available.

^a^ Nonrandomized trial.

^b^ Randomized clinical trial.

#### Postconsult Surveys to Examine Avoided Referrals

We found 25 studies that examined avoided referrals (Table 4), with 23 solely relying on a postconsult survey^2,3,13,15,17,18,20,21,22,24,25,26,28,33,39,41,42,43,45,50,52,55,59,75,77^ and 2 studies combining a postconsult survey with the recorded hospital visits.^2,50^ The percentage of avoided referrals varied from 24.0% (7277 of 30 320 referrals) to 55.0% (217 of 394 referrals). The percentage of extra referrals varied from 0% to 8.0% (22 of 94 referrals).

**Table 4.** The 4 Scenarios of Referrals for Studies Using Postconsult Surveys in Alphabetical Order

Article	No. of patients/No. of electronic consultations, (%)			
	Avoided referral	Referral still needed	Referral still not needed	Extra referral
Archibald et al,^13^ 2018	52/169 (30.8)	46/169 (27.8)	59/169 (35.5)	2/169 (1.2)
Chang et al,^15^ 2020	99/212 (47.0)	55/212 (26.0)	44/212 (21.0)	12/212 (6.0)
Fogel et al,^17^ 2017	200/436 (46.1)	108/436 (24.8)	88/436 (20.4)	17/436 (3.9)
Guglani et al,^18^ 2022	32/64 (51.3)	12/64 (19.4)	13/64 (21.7)	0
Hadden et al,^20^ 2022	105/406 (26.1)	186/406 (45.9)	84/406 (20.9)	12/406 (3.0)
Helmer-Smith et al,^21^ 2020	383/1064 (36.0)	202/1064 (19.0)	361/1064 (34.0)	21/1064 (2.0)
Johnston et al,^50^ 2017	110/276 (40.0)	41/276 (15.0)	93/276 (34.0)	22/276 (8.0)
Keely et al,^3^ 2013	255/594 (43.0)	112/594 (19.0)	172/594 (29.0)	17/594 (3.0)
Lai et al,^22^ 2018	5176/14 105 (36.7)	3794/14 105 (26.9)	4175/14 105 (29.6)	437/14105 (3.1)
Liddy et al,^25^ 2018	3598/9087 (39.6)	2235/9087 (24.6)	2544/9087 (28.0)	318/9087 (3.5)
Liddy et al,^24^ 2017	120/301 (40.0)	54/301 (18.0)	96/301 (32.0)	15/301 (5.0)
Liddy et al,^52^ 2019	25 822/60 474 (42.7)	14 695/60 474 (24.3)	16 327/60 474 (27.0)	1390/60 474 (2.3)
Liddy et al,^26^ 2019	44/111 (40.0)	35/111 (32.0)	19/111 (18.0)	4/111 (4.0)
Liddy et al,^28^ 2022	518/1016 (51.0)	193/1016 (19.0)	213/1016 (21.0)	30/1016 (3.0)
Muris et al,^2^ 2020	1885/3872 (48.7)	534/3872 (13.8)	1258/3872 (32.5)	193/3872 (5.0)
Nabelsi et al,^33^ 2019	90/225 (40.0)	51/225 (23.0)	65/225 (29.0)	9/225 (4.0)
Rostom et al,^39^ 2018	15/40 (38.0)	13/40 (34.0)	8/40 (21.0)	1/40 (3.0)
Russell et al,^59^ 2021	216/394 (55.0)	122/394 (31.0)	55/394 (14.0)	0
Shehata et al,^75^ 2016	21/62 (34.3)	11/62 (18.5)	25/62 (41.9)	0
Singh et al,^41^ 2021	92/289 (32.0)	46/289 (16.0)	130.05/289 (45.0)	5/289 (2.0)
Singh et al,^42^ 2022	7276/30 320 (24.0)	1516/30 320 (5.0)	17 585/30 320 (58.0)	0
Singh et al,^43^ 2022	561/1080 (52.0)	205/1080 (19.0)	237/1080 (22.0)	32/1080 (3.0)
Walker et al,^77^ 2020	84/302 (28.0)	75/302 (25.0)	NA	NA
Wang et al,^55^ 2021	125/329 (38.0)	52/329 (16.0)	141/329 (43.0)	0
Witherspoon et al,^45^ 2017	66/190 (35.0)	70/190 (37.0)	32/190 (17.0)	15/190 (8.0)

Abbreviation: NA, not available.

## Discussion

### Main Findings

This systematic review found that the most common reasons for implementing digital interdisciplinary e-consultation between FPs and hospital specialists were improving access to care and avoiding unnecessary referrals toward the hospital. Our systematic review shows that the majority of studies investigating digital interdisciplinary e-consultation were observational and most of the studies were performed in North America. The results of the studies indicate that the implementation of e-consultation is associated with improved access to hospital care and the avoidance of hospital referrals.

Previous systematic reviews^7,8^ reported a narrative synthesis of literature on population health, per capita health care costs, and both patient and clinician experience of care. These previous reviews^7,8^ showed that the majority of new research examined outcomes associated with patient and clinician experience, with stakeholder enthusiasm for e-consultations being high, and concluded that e-consultations do not appear to greatly increase the risk of sentinel events. An increased international presence of e-consultation and the breadth of specialty services offered has greatly expanded with most studies focusing on multispecialty services. Reviews also showed e-consultations provided faster access to specialists’ advice, resulting in substantial avoidance of face-to-face referrals. Liddy et al^7^ reported avoided face-to-face visits between 7.4% and 78% with most reporting between 22% and 68%. Furthermore, Liddy et al^7^ reported median response times from fewer than 1 days to fewer than 6 days, and Vimalananda et al^8^ reported response times of a few days or less. Since the latest systematic review^8^ reporting on literature up until February 2019, research on e-consultation has continued. The COVID-19 pandemic has accelerated the use of digital health tools.^82^ With previous reviews reporting a narrative synthesis of their findings, we chose to quantitatively report our findings to provide policymakers and health systems leaders with an overview of how e-consultation is associated with access to care and the avoidance of hospital referrals.

Avoiding hospital referrals helps to control health care costs and reduce unnecessary referrals, which is particularly helpful for patients unable to travel to the hospital or in times when a hospital visit is unwanted (eg, during the COVID-19 pandemic). The implementation of e-consultation might serve as a pull factor (ie, the supply of care [the e-consultation in this case] leads to an increased demand for care). The e-consultation might also lead to extra referrals to hospital specialists. Although these extra referrals are an added cost, it is unclear if these extra referrals are an undesirable outcome in terms of patient health. Long-term use of e-consultation might lead to a learning effect for FPs, especially when these e-consultations are used for training purposes, meaning that over time, FPs might refer less or refer more directly, influencing this referral percentage.

In the future, using nonrandomized trial designs and choosing appropriate outcomes can improve the strength of studies. Since the COVID-19 pandemic, use of digital health care has increased,^83^ showing the need for this kind of digital tool. Numerous studies^84,85,86^ have shown that all stakeholders are happy with the implementation of e-consultations. RCTs are impractical and costly, and therefore are often not included as part of the implementation of an electronic health tool. For e-consultation, an RCT would mean that several FPs do not gain access to e-consultation, which is often undesirable. One might question how important the strength of evidence is for policymaking. Despite the lack of evidence from RCTs, the use of e-consultation has greatly increased over the years, and other types of research (eg, observational research) can provide useful insights. Observational research is a quick and easy way to get a first impression of the effect of a digital tool, which can support policymakers in their decision. However, it is important to keep in mind that more high-quality studies should be performed before drawing a definite conclusion about the effect size.

### Limitations

This study has some limitations. We classified the GRADE scores of the outcome measurements in this review as either low or very low, indicating that an estimate of effect size is very uncertain, and that further research is needed to estimate the effect. Studies showed a great degree of heterogeneity even between RCT’s and nonrandomized trials. This heterogeneity was largely due to large differences in the number of e-consultations, and, thus, the standard error, across different studies. Therefore, estimating the size of effect on outcomes, and, thus, generalizing the findings by performing a meta-analysis remains difficult. Moreover, the absence of publication bias could not be guaranteed. These limitations make it difficult to draw a definite conclusion. As previous reviews suggested,^7,8^ nonrandomized trial designs are suited for research on e-consultations, which would improve the strength of the study. Furthermore, definitions of wait time across studies were poorly stated, and outcomes associated with access to care were reported as both medians and means. Because the data were most likely not normally distributed, reporting the median would be most appropriate.

Studies on e-consultation have mostly been performed in the US and Canada. The role of the FP in health care systems across countries differs. In some countries (eg, UK and the Netherlands) or parts of countries (eg, certain provinces in Canada), the FP has the role of gatekeeper, where the FP is responsible for determining who is referred to the hospital or not. Studies report on the implementation of e-consultation across a wide variety of specialties. Some specialties might be less suited for e-consultation, which might be due to the nature of the specialty. For example, general medical specialties might be better suited for e-consultation compared with surgical specialties. Other factors that might contribute to referral patterns are the type of health insurance used in a country, the distance from a hospital for the patient, the reimbursement of specialists for responding to e-consultation, and the workload of FPs and hospital specialists. Additionally, the implementation process, setting and users may have differed between studies (eg, a setting where the e-consultation was actively implemented and had lots of different hospital specialists able to respond may have led to shorter response times). These differences between studies might account for differences in outcomes.

## Conclusion

In this systematic review of the association of e-consultation with access to hospital care and the avoidance of hospital referrals, we found that the use of e-consultation has greatly increased over the years. Although e-consultation is associated with improving access to hospital care and avoiding hospital referrals, it is hard to draw a definitive conclusion about these outcomes due to heterogeneity and lack of high-quality evidence (ie, from RCTs). Nevertheless, e-consultation seems to be a promising digital health care implementation, but more rigorous studies are needed. We recommend using nonrandomized trial designs and choosing appropriate outcomes in future research on this topic.

## Notes

Supplement 1. **eAppendix 1.** Systematic Review Protocol **eAppendix 2.** Included Outcome Measurements **eAppendix 3.** Quality Assessment per Outcome Measurement: GRADE Scores

Supplement 2.
Data Sharing Statement
