# Supplementary material for: Family Physician–to–Hospital Specialist Electronic Consultation and Access to Hospital Care: A Systematic Review
Source: JAMA Netw Open. 2024 Jan 12;7(1):e2351623. doi: 10.1001/jamanetworkopen.2023.51623 (PMC10787322; doi:10.1001/jamanetworkopen.2023.51623)
Supplement: Supplement 1. — eAppendix 1. Systematic Review Protocol eAppendix 2. Included Outcome Measurements eAppendix 3. Quality Assessment per Outcome Measurement: GRADE Scores [file jamanetwopen-e2351623-s001.pdf]

Supplemental Online Content

Peeters KMM, Reichel LAM, Muris DMJ, Cals JWL. Family physician-to-hospital specialist electronic consultation and access to hospital care: a systematic review. *JAMA Netw Open*. 2024;7(1):e2351623. doi:10.1001/jamanetworkopen.2023.51623

eAppendix 1. Systematic Review Protocol

eAppendix 2. Included Outcome Measurements

eAppendix 3. Quality Assessment per Outcome Measurement: GRADE Scores

This supplemental material has been provided by the authors to give readers additional information about their work.

© 2024 Peeters KMM et al. *JAMA Network Open.*

eAppendix 1. Systematic Review Protocol

**Rationale**	Previous systematic reviews analyzed evidence on family physician (FP) to medical specialist e-consultations using a narrative synthesis approach including an overview of its impact on population health. Yet from the perspective of the two persons in the FP's consultation room – the FP and the patient – there are a few outcomes most relevant when contemplating using an e-consultation to contact a medical specialist. Does it provide the patient with timely access to hospital care? Does it enable the FP to (continue to) provide appropriate and well-informed care without the patient needing to visit the hospital outpatient department?
**Objective(s)**	The focus of this review is to provide a quantitative synthesis of these outcomes; access to hospital care and the avoidance of hospital referrals. We aim to answer the following questions: 1) What is the effect of FP-hospital specialist e-consultation on access to care and the avoidance of hospital referrals? 2) What is the quality of the evidence regarding these outcome measures?
**Eligibility criteria**	We only included original research papers and therefore excluded opinion papers, policy papers, guidelines, pre-prints, protocols, reviews, notes, editorials, letters, and abstracts. Furthermore, we excluded studies evaluating e-consultations that did not fit our previously mentioned definition, e.g patient-doctor e-consultations, unsolicited e-consultations, video-consultations, e-mail consultations, one-way consultations, patient-provider modalities, electronic referrals, online discussion forums, and studies that did not study the effects of e-consultations separately. We also excluded studies about teledermatology, since we

© 2024 Peeters KMM et al. *JAMA Network Open.*

	view this as a stand-alone type of consultation, and multiple reviews have reported on outcomes of the specific service. We excluded studies that did not contain any of the outcome measurements on access to care or the avoidance of hospital referrals.
**Information source(s)**	Pubmed, Medline and Embase. Reference lists of screened articles.
**Study records:**<br>**- Data management**<br>**- Selection process**<br>**- Data collection process**	Two independent reviewers will screen titles/abstracts using Covidence in the first screening. A group consensus meeting will be held to check for differences. After this first round, the same two independent reviewers will screen the full text of the articles, after which another group consensus meeting will be held. Data collection and management will be done using Microsoft Excel.
**Outcomes**	Response time. Time spent answering e-consultation. Wait time. Referrals.
**Risk of bias**	To assess for risk of bias, GRADE scores will be assigned on outcome level.
**Data synthesis**	Outcomes will be quantitatively synthesized in tables. The Meta-Essentials package (version 1.4) will be used to explore consistency (e.g. I^2) and if appropriate, data will be synthesized in a forest plot. If appropriate, sensitivity and subgroup analysis will be done (e.g. per country).
**Meta-bias(es)**	Publication bias will be assessed using a funnel plot.
**Confidence in cumulative evidence**	According to GRADE scores on outcome level.

Search strategy for PubMed, Medline and Embase

© 2024 Peeters KMM et al. *JAMA Network Open.*

PubMed/Medline

((electronic[ti] AND consultation*[ti]) OR (remote[ti] AND consultation*[ti]) OR (electronic[ti] AND referral*[ti]) OR (e-consultation*[ti]) OR (e-referral*[ti])) OR (("Remote Consultation"[Majr] AND (e-consultation*[tiab] OR e-referral*[tiab] OR electronic consultation*[tiab] OR electronic referral*[tiab])) OR ("Electronic Consultation"[Majr] AND (e-consultation*[tiab] OR e-referral*[tiab] OR remote consultation*[tiab] OR electronic referral*[tiab]))OR ("e-consultation"[Majr] AND (electronic consultation*[tiab] OR e-referral*[tiab] OR remote consultation*[tiab] OR electronic referral*[tiab])) OR ("e-referral "[Majr] AND (electronic consultation*[tiab] OR e-consultation*[tiab] OR remote consultation*[tiab] OR electronic referral*[tiab])) OR ("Electronic referral "[Majr] AND (electronic consultation*[tiab] OR e-consultation*[tiab] OR remote consultation*[tiab] OR e-referral*[tiab])) OR ("Electronic Health Records"[Majr] AND (e-consultation*[tiab] OR e-referral*[tiab] OR remote consultation*[tiab] OR electronic referral*[tiab] OR electronic consultation*[tiab])))

Embase

('electronic consultation'/exp OR 'remote consultation'/exp OR 'e-consultation'/exp OR 'electronic referral'/exp OR 'e-referral'/exp) OR ('teleconsultation'/exp OR 'telemedicine'/exp OR 'telehealth'/exp OR 'remote'/exp AND ('consultation'/exp OR 'referral'/exp)) OR ('electronic health record'/exp AND ('consultation'/exp OR 'referral'/exp))

© 2024 Peeters KMM et al. *JAMA Network Open.*

eAppendix 2. Included Outcome Measurements

Themes	Outcome measurements	Definition
**Access to care**	Response time	Time between submission by FP and response by a specialist.
	Time spent answering e-consultation	Time between opening and closing of the consult by a specialist.
	Wait time	Time between submission of e-consultation and response by a specialist <u>or</u> hospital visit by a patient.
**Hospital referrals**		The number of patients that would normally have been referred to the hospital but were not referred due to FP use of e-consultation.<br>Avoided hospital visits can be calculated based on different data:<br>1) Based on direct measurement of actual hospital visits,<br>2) Based on referrals as reported by FPs,<br>3) Based on post-consult surveys completed by FPs,<br>4) Based on referral recommendations as indicated by hospital specialists.

© 2024 Peeters KMM et al. *JAMA Network Open.*

eAppendix 3. Quality Assessment per Outcome Measurement: GRADE Scores

Outcome measure	Design	GRADE-score design	GRADE-score outcome measure
Avoided referrals (n = 57)	RCT (n = 2)	Moderate	Low
	Quasi-experimental (n = 4)	Low	
	Observational (n = 51)	Very low	
Post-consult survey (n = 25)	RCT (n = 0)	/	Very low
	Quasi-experimental (n = 0)	/	
	Observational (n = 25)	Very low	
Response time (n = 48)	RCT (n = 1)	Low	Very low
	Quasi-experimental (n = 3)	Low	
	Observational (n = 44)	Very low	

© 2024 Peeters KMM et al. *JAMA Network Open.*

Time spent answering<br><br>(n = 16)	RCT<br><br>(n = 0)<br><br>Quasi-experimental<br><br>(n = 0)<br><br>Observational<br><br>(n = 16)	/<br><br><br><br>/<br><br><br><br>Very low	Very low
Wait time<br><br>(n = 11)	RCT<br><br>(n = 1)<br><br>Quasi-experimental<br><br>(n = 2)<br><br>Observational<br><br>(n = 8)	Low<br><br><br><br>Low<br><br><br><br>Very low	Very low

© 2024 Peeters KMM et al. *JAMA Network Open.*
