# Supplementary material for: Family Physician–to–Hospital Specialist Electronic Consultation and Access to Hospital Care: A Systematic Review
Source: JAMA Netw Open. 2024 Jan 12;7(1):e2351623. doi: 10.1001/jamanetworkopen.2023.51623 (PMC10787322; doi:10.1001/jamanetworkopen.2023.51623)
Supplement: Supplement 2. — Data Sharing Statement [file jamanetwopen-e2351623-s002.pdf]

# Data Sharing Statement

Peeters. Family Physician-to-Hospital Specialist Electronic Consultation and Access to Hospital Care. *JAMA Netw Open*. Published January 12, 2024.
doi:10.1001/jamanetworkopen.2023.51623

**Data**
**Data available:** No
